# Computed tomography analysis of glenoid size and bone depth: insights for baseplate fixation in reverse shoulder arthroplasty

**DOI:** 10.1016/j.xrrt.2026.100846

**Published:** 2026-08-06

**Authors:** Alexandre Quemener-Tanguy, Sami El Ramadan, Gauthier Menu, Etienne Boyer, Sébastien Aubry, François Loisel, Laurent Obert

**Affiliations:** a Orthopedic, Traumatology, Plastic and Hand Surgery Unit & CIC IT 808 University Hospital of Besancon, Besançon, France; b Radiology Department, CHU de La Réunion, Saint-Denis, France; c Clinique Saint Vincent, CHU de La Réunion, Saint-Denis, France; d Clinique épaule, Bordeaux, France; e Radiologie Department, Clinique Saint Vincent, Besançon, France

**Keywords:** Shoulder, Glenoid size, Reverse total shoulder arthroplasty, Computed tomography, Classification, Implant fixation, Pre-operative planning

## Abstract

**Background:**

Glenoid morphology plays a critical role in reverse total shoulder arthroplasty, influencing implant positioning and fixation. Although advanced Three dimensional pre-operative planning allows patient-specific optimization, a simple and reproducible framework for rapid assessment of glenoid size and bone stock remains lacking. The purpose of this study is to propose a computed tomography-based classification of glenoid size using a reproducible parameter and evaluate its potential as a complementary screening tool for pre-operative planning.

**Methods:**

A retrospective analysis of 200 non-pathological glenoids was performed. Measurements included maximum anteroposterior diameter (MAD, representing glenoid width), superoinferior diameter, surface area, and glenoid bone depth. A K-means clustering algorithm based on MAD defined 3 groups. Correlations with patient height were assessed. Baseplate compatibility and fixation potential were evaluated theoretically.

**Results:**

Three groups were identified: small (MAD <27 mm), medium (27-30 mm), and large (>30 mm). MAD strongly correlated with glenoid surface area (r = 0.82, *P* < .001). A 24-mm baseplate provided full bone contact in 90% of cases. Glenoid bone depth increased significantly with glenoid size (*P* < .001), supporting adaptation of screw length and positioning.

**Conclusion:**

This classification provides a simple and reproducible anatomical framework for glenoid size stratification. While not intended to replace patient-specific three dimensional planning, it may serve as a complementary screening tool to anticipate implant sizing and fixation strategies. Validation in pathologic glenoids is required.

Glenoid morphology is a key determinant of implant positioning, fixation, and outcomes in reverse total shoulder arthroplasty (rTSA).^3^^,^^5^^,^^23^ Stable baseplate fixation requires adequate bone contact and sufficient subchondral bone stock for screw anchorage.^6^^,^^12^ While loosening remains relatively uncommon, it can lead to complex revision procedures with significant bone loss.^19^^,^^22^

Advances in three dimensional (3D) pre-operative planning, navigation, and augmented reality have improved surgical accuracy.^10^^,^^20^^,^^21^ Although patient-specific 3D planning software is increasingly accessible, its routine use varies widely across centers, healthcare systems, and resource settings. Even when available, such tools do not eliminate the value of a rapid, computed tomography (CT)-based anatomical framework for initial assessment and educational purposes.

Previous studies have highlighted substantial variability in glenoid morphology.^3^^,^^5^^,^^23^ Imaging-based analyses using both CT and magnetic resonance imaging have confirmed this variability, particularly in glenoid dimensions such as width and height.^9^ However, most remain descriptive and lack direct integration into surgical workflows.

Establishing a classification based on native glenoid anatomy may provide a useful baseline for understanding variability and anticipating surgical constraints.^14^^,^^15^ Such a framework could support screening, education, and pre-operative planning.

The primary objective of this study was to define a simple 3-category classification of glenoid size based on CT measurements. Secondary objectives included assessing correlations with patient height, evaluating baseplate compatibility, and analyzing glenoid bone depth distribution. We hypothesized that maximum anteroposterior diameter (MAD) would serve as a reliable and reproducible surrogate for glenoid size stratification and that glenoid bone depth would vary systematically across size groups, with implications for baseplate fixation planning.

## Materials and methods

This is a retrospective cross-sectional anatomical study of 200 non-arthritic glenoids, selected from patients who underwent thoracic or polytrauma CT scanning for indications unrelated to shoulder pathology. Exclusion criteria included shoulder pathologies (osteoarthritis, tumor, fracture, or rotator cuff tear) or missing height data. Demographic data (age, sex, height, and side) were collected. CT scans were performed using a Philips Brilliance CT (Eindhoven, Netherlands) or Siemens Somatom Sensation CT (Erlangen, Germany). Measurements were performed using the Kodak Carestream PACS System (Rochester, NY, USA) with multiplanar reformatting.

**Figure 1:**
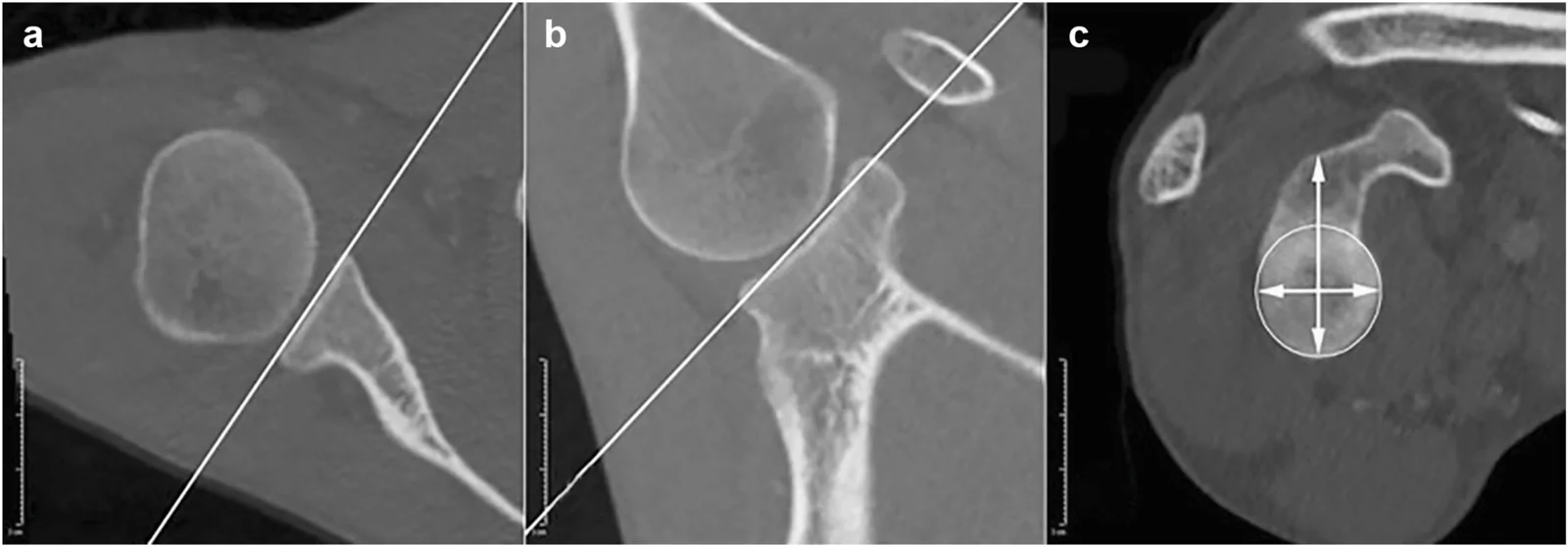
Multiplanar orientation through the articular surface of the glenoid in the axial (**a**) and coronal (**b**) planes used to determine the glenoid’s long axis. Glenoid parameters measured on the reference slice (**c**).

**Figure 2:**
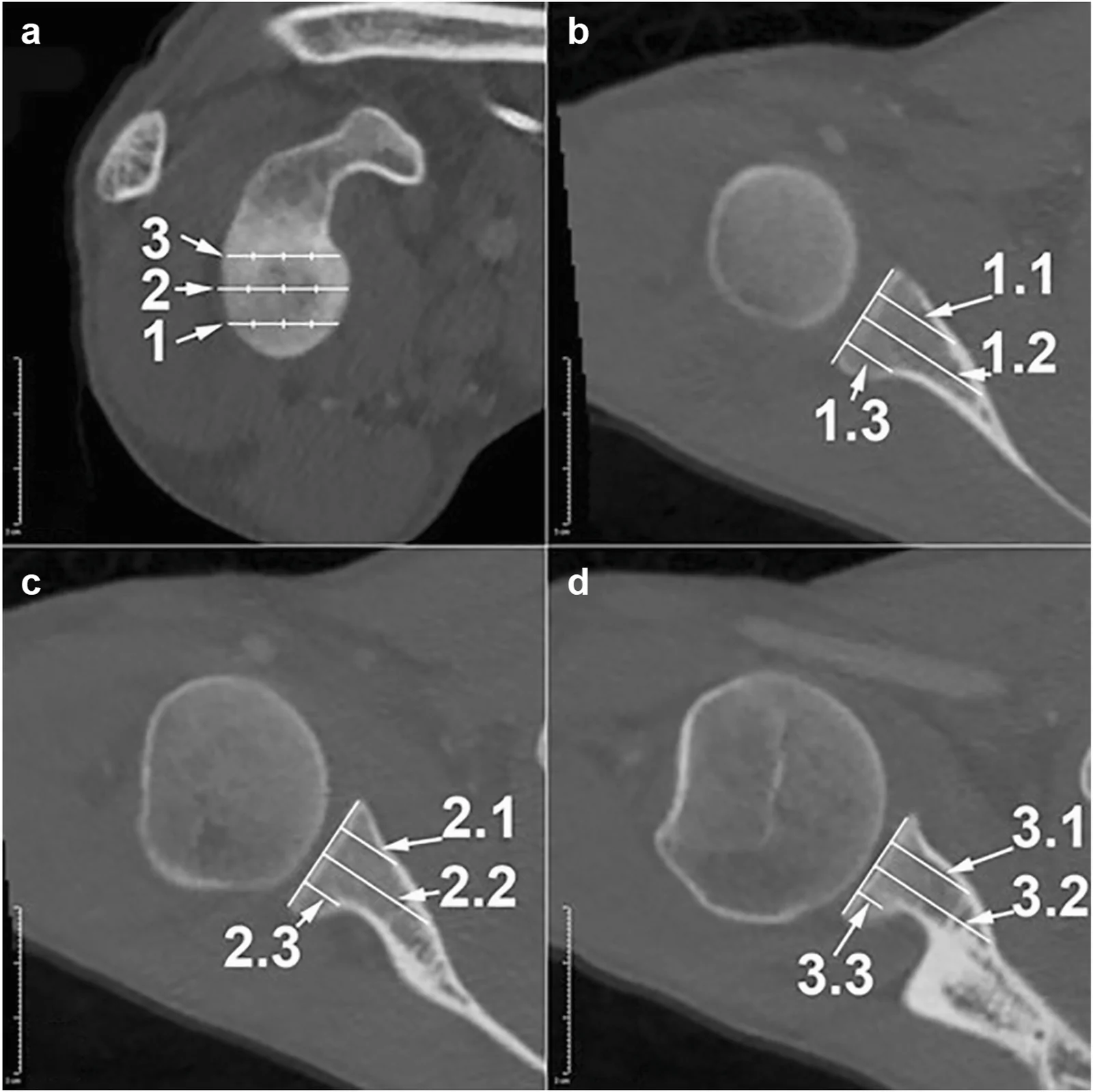
Bone stock measurement: reference slice of the glenoid (**a**). Three equatorial lines (1, 2, 3) divide the articular surface into 4 equal parts. Perpendicular slices to the reference plane passing through lines 1, 2, and 3, respectively (**b-d**). Bone stock measured at the following points: infero-anterior (1.1), infero-central (1.2), infero-posterior (1.3), mid-anterior (2.1), mid-central (2.2), mid-posterior (2.3), supero-anterior (3.1), supero-central (3.2), and supero-posterior (3.3).

**Figure 3:**
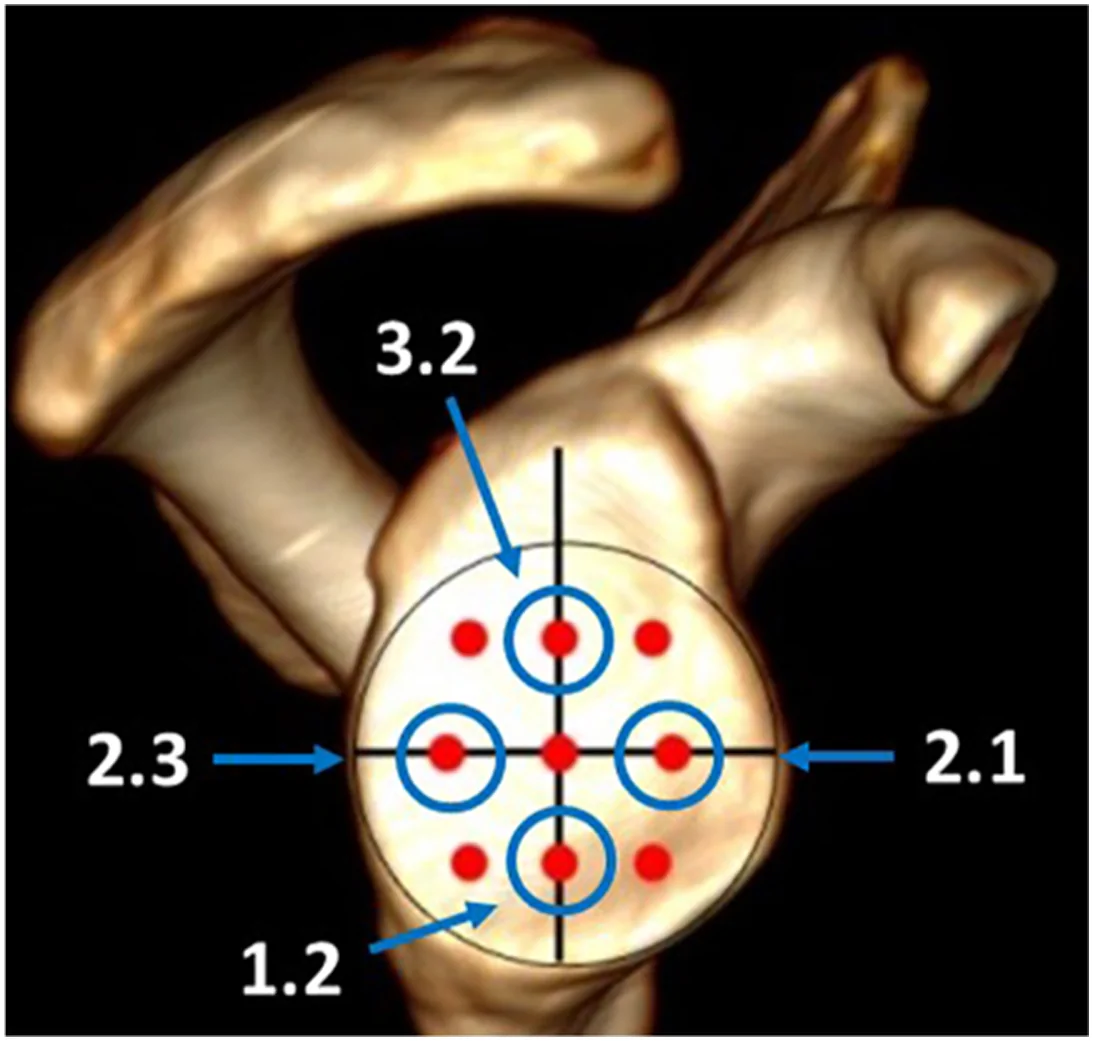
Screw fixation positions of the baseplate.

The slices were standardized by selecting an axial slice perpendicular to the glenoid articular surface plane, at the level at which the coracoid process was visible, to ensure reproducibility (Fig. 1). This level was chosen because it reliably corresponds to the center of the glenoid face. The orientation perpendicular to the glenoid surface was preferred over a scapular-axis reference because it directly reflects the implant seating plane and has been shown to be more reproducible in prior studies. The measured parameters were MAD, measured in millimeters (mm) on sagittal slices; maximum superoinferior diameter (SID), measured in mm on sagittal slices; glenoid surface area, calculated in mm^2^ from a circle tangent to the inferior glenoid rim^4^; and glenoid bone depth (defined as the depth of cancellous bone available for screw anchorage, measured perpendicular to the articular surface), measured at 9 points (Fig. 2), with 4 key points retained (3.2 superior, 2.1 anterior, 2.3 posterior, and 1.2 inferior) corresponding to the screw fixation positions of the baseplate (Fig. 3). MAD was defined as the MAD (glenoid width). Glenoid bone depth was measured on the axial multiplanar reconstruction plane from the glenoid articular surface cortex to the far cortex of the glenoid vault, perpendicular to the articular surface at each of the 9 predefined points. These measurements represent the maximum available bone depth for screw–anchorage under ideal conditions, without accounting for reaming depth, baseplate thickness, or augmentation techniques (eg, bony increased offset–reverse shoulder arthroplasty or medialized implant options), which may substantially reduce achievable screw lengths in clinical practice.

To assess interobserver and intraobserver reproducibility, the various measurements were performed by 1 radiologist and 3 shoulder surgeons, allowing calculation of intraclass correlation coefficients (ICCs). Continuous variables were expressed as mean ± standard deviation after checking for normality using the Shapiro Wilk test. Correlations between glenoid dimensions and patient height were evaluated using Pearson's correlation coefficient. The MAD was selected as the reference parameter for classification due to its excellent interobserver and intraobserver reproducibility and its strong correlation with glenoid surface area. A K-means clustering algorithm was applied to define 3 groups, as this number was considered clinically relevant and allowed for a simple and practical classification usable in surgical planning. The choice of k = 3 was validated using the elbow method on the within-cluster sum of squares, showing an inflection point at k = 3. Given the number of correlations tested, a Benjamini-Hochberg correction for multiple comparisons was applied to control the false discovery rate. Precise *P* values are reported throughout; a threshold of *P* < .05 was used for statistical significance.

**Figure 4:**
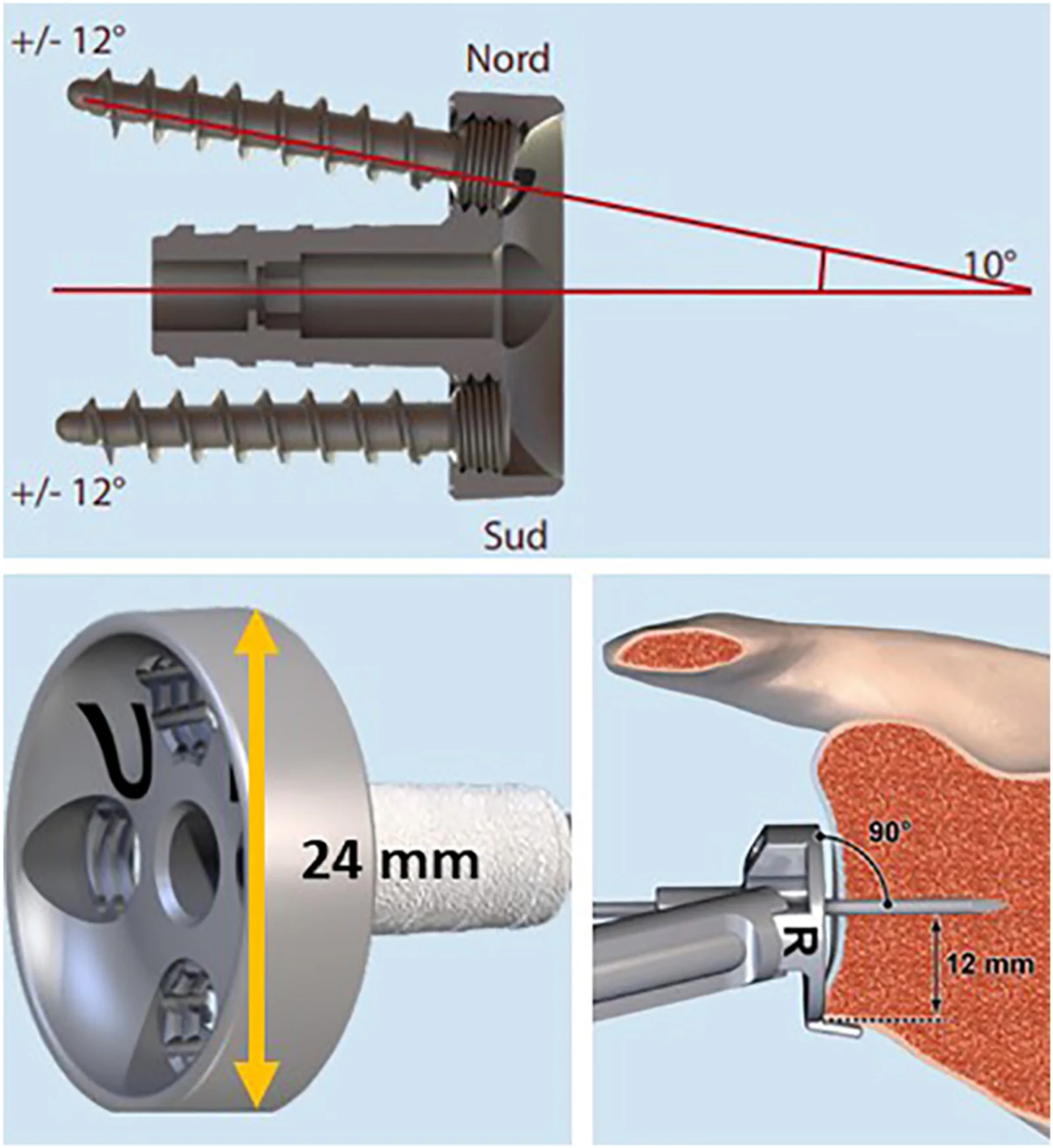
Baseplate with a 24-mm diameter.

To determine the percentage of bone-baseplate contact, we used the surface area of a 24-mm baseplate (Fig. 4) and those of various implants available on the market, comparing them to the glenoid surface areas in the 3 defined groups. Baseplate compatibility was assessed theoretically by comparing the surface area of commercially available baseplates with measured glenoid surface areas. No implants were physically tested or implanted. The study followed ethical standards for retrospective analysis and received institutional review board approval (IRB-SOFCOT-15-2025).

## Results

The cohort included 200 glenoids (116 female, 84 male; sex ratio 1.38), with a mean patient age of 65 ± 15 years and a mean height of 167 ± 10 cm. The mean measurements were MAD 27.6 ± 3.3 mm, SID 38.5 ± 5.1 mm, and glenoid surface area 625.5 ± 139.5 mm^2^. Significant correlations were observed between patient height and MAD (r = 0.54, *P* < .001), SID (r = 0.34, *P* < .001), and glenoid surface area (r = 0.57, *P* < .001). Significant correlations were also found between glenoid dimensions: MAD showed a strong correlation with glenoid surface area (r = 0.82, *P* < .001), SID correlated with glenoid surface area (r = 0.68, *P* < .001), and MAD showed a moderate correlation with SID (r = 0.49, *P* < .001).

For interobserver reproducibility, ICCs were 0.99 for MAD (*P* < .001), 0.91 for SID (*P* < .001), and 0.88 for surface area (*P* < .001). For intraobserver reproducibility, ICCs were 0.98, 0.94, and 0.91, respectively (all *P* < .001). MAD was selected as the basis for classification due to its high reproducibility and strongest correlation with glenoid surface area.

The K-means algorithm identified 3 groups: small glenoids (MAD <27 mm, n = 76, mean MAD = 24.5 ± 1.4 mm), medium glenoids (MAD 27–30 mm, n = 88, mean MAD = 28.5 ± 1.2 mm), and large glenoids (MAD >30 mm, n = 36, mean MAD = 32.6 ± 2.7 mm). A 24-mm baseplate (surface area = 452 mm^2^) provided complete bone-baseplate contact in 90% of glenoids. The remaining 10%, all of whom had a MAD <24 mm, demonstrated incomplete coverage, with a mean bone-baseplate contact of 98.8% in this subgroup. These glenoids all belonged to the small group and were predominantly female patients (mean MAD = 22.1 ± 1.1 mm, mean height = 158 ± 7 cm). No incomplete contact was observed in medium or large glenoids. Comparison with other baseplates available on the market is shown in Table I.

**Table I:** Bone–baseplate contact for commercially available baseplates in our cohort.

Medical device companies	Model	MAD (mm)	Glenoid with full bone–baseplate contact (%)
Arthrex	Univers Revers, Modular Glenoid System	24/28	90/51
DePuy Synthes	Delta Xtend	27	61
Evolutis	Unic	26/30	71/29
FH Ortho	Arrow II	25.5/28.5/31.5	71/51/8
FX solutions	Humelock/Easytech	24	90
Lépine	Aramis	25/28	84/51
Move up	ISA	26	71
Medacta	Medacta Shoulder System	22/24.5/27	91/90/61
Stryker	Aequalis Reversed II	25/29	84/38
Zimmer Biomet	Comprehensive Reverse Shoulder System	25/28	84/51

*MAD*, maximum anteroposterior diameter.

MAD values correspond to available baseplate diameters for each implant system. Percentages represent the proportion of glenoids in our cohort achieving full theoretical bone–baseplate contact for each size.

Bone depth increased with glenoid size (*P* < .001), as detailed in Table II. Screw lengths of 20-25 mm for point 3.2 (superior), 15-20 mm for point 1.2 (inferior), 10 mm for point 2.1 (anterior), and 10-15 mm for point 2.3 (posterior) would be compatible with most glenoids. Additionally, male patients had significantly larger glenoids (mean MAD = 29.7 ± 3.1 mm) than female patients (mean MAD = 25.8 ± 2.7 mm) (*P* < .001). Women represented 84.2% of the small glenoid group.

**Table II:** Glenoid bone depth according to glenoid size.

Bone depth measurement points	Small glenoid (<27 mm)	Medium glenoid (27-30 mm)	Large glenoid (>30 mm)	Pearson correlation coefficient	*P* value
3.2 (superior) mean (SD), [min-max]	21.5 (4.3) [12-31]	23.1 (4.9) [9-39]	27.3 (5.7) [21-54]	r = 0.37	<.001
2.1 (anterior) mean (SD), [min-max]	8.9 (1.9) [5-13]	10.4 (5.1) [6-38]	10.4 (2.2) [6-15]	r = 0.17	.018
2.3 (posterior) mean (SD), [min-max]	12.3 (5.5) [5-34]	15 (8.1) [4-49]	18.6 (11.8) [8-50]	r = 0.26	<.001
1.2 (inferior) mean (SD), [min-max]	13.9 (5.1) [7-28]	18.5 (8.1) [7-41]	18.7 (9.3) [5-42]	r = 0.26	<.001

*SD*, standard derivation.

## Discussion

The main finding of this study is that a simple CT-based parameter, the MAD, enables reproducible stratification of glenoid size into 3 groups associated with distinct glenoid bone depth characteristics. These findings suggest that glenoid width may serve as a practical surrogate for estimating bone stock and anticipating fixation constraints. However, this classification is not intended to replace patient-specific pre-operative planning, but rather to complement it by providing a simple and accessible anatomical framework.

Our results are consistent with previous anatomical studies. MAD showed the strongest correlation with glenoid surface area, in line with Moineau et al,^15^ and remains an easily identifiable and reproducible parameter on CT scans. Similar concerns regarding the limitations of patient height as a predictor of glenoid size have been raised by Verweij et al,^25^ reinforcing the relevance of imaging-based measurements. The distribution of glenoid sizes observed in our cohort is also comparable to that reported by Churchill et al,^3^ who described mean glenoid widths of approximately 29 mm and heights of 39 mm in a cadaveric series, with substantial sex-related variability, supporting the external validity of our findings. It should be noted that the strong correlation between MAD and glenoid surface area is in part mathematically expected, since glenoid surface area was estimated from a circle tangent to the inferior rim, making its diameter closely related to MAD. This relationship reinforces the choice of MAD as a surrogate but should not be interpreted as an independent novel finding.

From a technical perspective, glenoid bone depth varied according to glenoid size and location, highlighting potential constraints for screw fixation. Although the proposed screw trajectories are theoretical, they emphasize the importance of understanding regional bone stock when planning fixation. Previous cadaveric studies have demonstrated the risk of suprascapular nerve injury or subscapularis interference depending on screw positioning,^8^^,^^16^ supporting the need for careful pre-operative assessment. Our classification complements existing approaches, such as the Chandigarh system,^1^ by integrating quantitative clustering and bone depth analysis.

The analysis of commercially available baseplates showed that a 24-mm diameter implant achieved adequate bone contact in most cases, particularly in smaller glenoids. While full contact was not always achieved, prior biomechanical studies suggest that partial contact may still provide sufficient primary stability.^7^^,^^13^ These findings support the use of smaller baseplates in appropriate anatomical settings, although implant selection should remain individualized. In addition, matching baseplate size to glenoid morphology has been shown to improve stability and reduce micromotion,^2^ while other studies reported no significant differences in bone strain across implant configurations, supporting the use of smaller baseplates even in larger glenoids.^17^ Other factors influencing fixation include bone density, central peg design, and screw length,^12^ reinforcing the importance of detailed anatomical assessment.

Clinically, this classification may be most directly applicable in settings where glenoid deformity is limited and 3D pre-operative planning is not systematically performed—most notably in rTSA for proximal humeral fracture, where glenoid anatomy is generally preserved and surgery is often performed in an acute context. In these cases, the classification could support rapid implant sizing and fixation planning based on a single CT measurement. It may also serve an educational role by helping surgeons anticipate anatomical variability before undertaking rTSA. Given that sex-related differences in shoulder arthroplasty outcomes and implant considerations have been reported,^18^^,^^24^ such an approach may be particularly relevant when assessing smaller glenoids. Nevertheless, modern tools such as 3D planning, navigation, and augmented reality remain essential to optimize implant positioning and should be considered complementary to this approach.^10^^,^^11^^,^^20^^,^^21^

This study has several strengths, including a large sample size, standardized CT-based measurements, and the use of an objective clustering method. However, several limitations must be acknowledged. The use of nonpathological glenoids limits direct clinical extrapolation, as pathologic glenoids often present deformity, bone loss, and altered version. In addition, screw trajectories were modeled without accounting for glenoid retroversion, implant orientation, or baseplate-specific design parameters. Fixation was assessed using a theoretical approach, without experimental or clinical validation, and important anatomical constraints such as the coracoid process and scapular pillar were not considered. Finally, the retrospective design and restriction to a single population may limit generalizability.

Future studies should evaluate this classification in pathologic glenoids and determine its clinical relevance in relation to implant positioning, fixation strength, and surgical outcomes.

## Conclusion

This study proposes a simple CT-based classification of glenoid size using MAD. It provides a reproducible anatomical framework that may assist in pre-operative assessment. It should be considered complementary to 3D planning rather than a replacement. Further validation in pathologic conditions is necessary.

## Disclaimers

Funding: No funding was disclosed by the authors.

Conflicts of interest: Laurent Obert reports fees from FX Shoulder Solutions, Elsevier, and Evolutis outside the sub-mitted work and royalties from FX Shoulder Solutions, Med-artis, Kerimedical, TBF, Evolutis, and Branchet. Any additional authors, their immediate families, and any research foundations with which they are affiliated have not received any financial payments or other benefits from any commercial entity related to the subject of this article.
